# Cycloprodigiosin: A multispecies settlement cue for scleractinian coral larvae

**DOI:** 10.1038/s41598-025-12409-5

**Published:** 2025-07-25

**Authors:** Laura J. Fiegel, Samuel Nietzer, David Brefeld, Robbert C. Geertsma, Ronald Osinga, Peter J. Schupp, Matthias Y. Kellermann

**Affiliations:** 1 Institute for Chemistry and Biology of the Marine Environment (ICBM), Carl-von-Ossietzky University Oldenburg, Schleusenstrasse 1, 26382 Wilhelmshaven, Germany; 2 Marine Animal Ecology, Wageningen University & Research, Droevendaalsesteeg 1, Wageningen, 6708 PB The Netherlands; 3 Helmholtz Institute for Functional Marine Biodiversity (HIFMB), Carl-von-Ossietzky University Oldenburg, Ammerländer Heerstrasse 231, 26129 Oldenburg, Germany

**Keywords:** Chemical settlement cues, Cycloprodigiosin, Scleractinian corals, Coral larval settlement, Reef restoration, Chemical ecology, Restoration ecology, Tropical ecology

## Abstract

The survival of coral reefs depends on the rejuvenation of coral populations with the potential to adapt and survive a changing climate. Assisted sexual reproduction has become an important tool in reef management. One bottleneck is the efficient and manageable induction of coral larval settlement. Here we report cycloprodigiosin (CYPRO) as a multispecies cue which induces complete larval settlement at rates between 40 and 93% in four brooding and five broadcast spawning species. All nine tested species showed similar uptake and conversion of CYPRO (photolytic degradation), which together initiate and complete the transition to successful settlement. Due to its chemical stability and low water solubility, the component can be easily stored, transported and applied on clean substrates, which might increase the chances of survival of the settled recruits. Thus, this broad settlement cue has the chance to advance reef restoration projects.

**Supplementary Information:**

The online version contains supplementary material available at 10.1038/s41598-025-12409-5.

## Introduction

Coral reefs are exceptional ecosystems that face a multitude of anthropogenic stressors such as overfishing, pollution, but foremost climate change and the associated increase in heat events^1^. Extreme heat over a prolonged period of time disrupts the essential symbiosis between coral hosts and their associated symbiotic algae^2^. The interrupted symbiosis can lead to mass bleaching events as globally witnessed in 2023-24 already for the fourth time^3^. Mass bleaching often results in large coral community die-offs and, associated with it, long-term ecosystem shifts^4,5^. To counteract this environmental crisis, methods have been developed to restore damaged reefs on a small scale. However, most of these methods rely on asexual reproduction through coral fragmentation and growth of these clones, that have the same characteristics as their parental colony. Juvenile corals obtained from sexual reproduction, in contrast, possess both a large genetic diversity as well as an inherent phenotypical plasticity, resulting in a higher adaptive potential^6^. Therefore, coral restoration procedures in recent years have extended from cloning via fragmentation towards the production of sexually reproduced coral generations^7–10^. The latest research showed that these sexually reproduced corals might be more tolerant to global warming^11^ and also retain the genetic diversity of natural coral populations^12^.

Scleractinian corals reproduce sexually in two distinct modes: brooding and broadcast spawning. Both reproductive strategies result in swimming coral larvae that need to detect a suitable place to settle. After attachment to a solid substrate, coral larvae metamorphose into a sessile coral recruit and cement their foundations by calcification towards a juvenile coral colony. This initial step of settlement on a specific substrate is driving the survival of young corals. The choice of settlement location is influenced by many environmental factors^10^ such as light spectra and intensity^13,14^reef sound^15^substrate colors^16,17^substrate microtopography^18^but is primarily triggered by biochemical cues that are produced by reef organisms such as crustose coralline algae (CCA^19–22^), and their associated microbiome^23,24^. Two methods are commonly used to promote the settlement of coral larvae during restoration measures: (1) establishment of CCA with their natural bacterial community on settlement substrates^25^ and (2) mature natural biofilms that cover substrates^8,9,26^. However, both methods have their limitations. With CCA, settlement rates can vary greatly depending on the coral and CCA species^27–29^. Also, the bacterial community of CCA is highly dynamic and shows sensitivity to environmental shifts such as warming^30^ or acidification of the ocean^31^. Once CCA are transferred from the reef into an aquarium facility, their microbial community may change^32^ and their ability to induce settlement of coral larvae may diminish over time. Some CCA species utilize competitive strategies such as overgrowth and sloughing, which can significantly reduce post-settlement survival rates of coral recruits^25,33^. By comparison, long incubated biofilms on suitable settlement tiles are independent of CCA but the established microbial communities (e.g., bacteria, microalgae, protists) strongly depend on the prevailing bacterial community of the seawater, the culturing time and the selected culturing conditions (i.e., temperature, light regime and nutrient composition)^34^. The established biofilm may also include harmful organisms, which could have a significant negative impact on post settlement survival^35–37^. Thus, settlement triggered by barely controllable biological systems is unpredictable and can vary greatly in its efficiency among batches.

In the attempt to decouple biological variability (i.e., CCA or microbial biofilms) in the fragile settlement reaction of coral larvae, isolated chemical agents are becoming increasingly recognized. In 2011, the compound tetrabromopyrrole (TBP) was isolated and identified from the CCA-associated bacterial strain *Pseudoalteromonas* sp. and found to induce settlement in multiple coral species^38–41^. The cnidarian neuropeptide Hym-248, member of the Glycine-Leucine-Tryptophan-amide family neuropeptides (GLW-amides), was found to be a highly species-specific settlement cue. This compound induces high settlement rates in Acroporids, but very low or no settlement in species of the genera *Dipsastrea*, *Favia*, *Lobophyllia*, *Pachyseris*, *Porites*, *Platygyra* and *Montipora*^36,42–44^. Other neurotransmitters such as Epinephrine and Dopamine also showed inductive capacity in coral larvae, with success rates of 35–40%, and these compounds were solely tested on the brooding species *Leptastrea purpurea*^45^. Another example of a chemical settlement inducer is the bromotyrosine derivative 11-deoxyfistularin-3, which was isolated from 15 kg of CCA rubble. Application of this compound as a singular settlement agent to larvae of *Pseudosiderastrea tayamai* only resulted in moderate settlement success rates of 28%, but settlement was enhanced up to almost 90% in combination with the carotenoid fucoxanthinol and/or fucoxanthin^46^. Most recently, the red pigment cycloprodigiosin (CYPRO), identified and isolated from the CCA-associated bacterial strain *Pseudoalteromonas rubra*^24,47^induced settlement in larvae of *L. purpurea* at an efficiency close to 90% of settlement success. A follow-up study, also using *L. purpurea* as test species, showed that chemically induced settlement with CYPRO followed the same development pathway compared to CCA induced settlement and that both strategies led to successful transformation of settlers into young adult colonies (observed > 1 year of age^48^).

In this study, we tested the settlement cue CYPRO on nine widespread coral species including four brooding species (i.e.,* Leptastrea purpurea*, *L. transversa* and *Pocillopora acuta* and *Favia fragum)* and five broadcast spawning species (i.e., *Acropora humilis*, *A. hemprichii*, *A. kenti*, *A. millepora* and *A. microclados*). Successful settlement across all species corroborate its potential as a multispecies settlement inducer with possible applications in reef restoration.

## Results

**Fig. 1:**
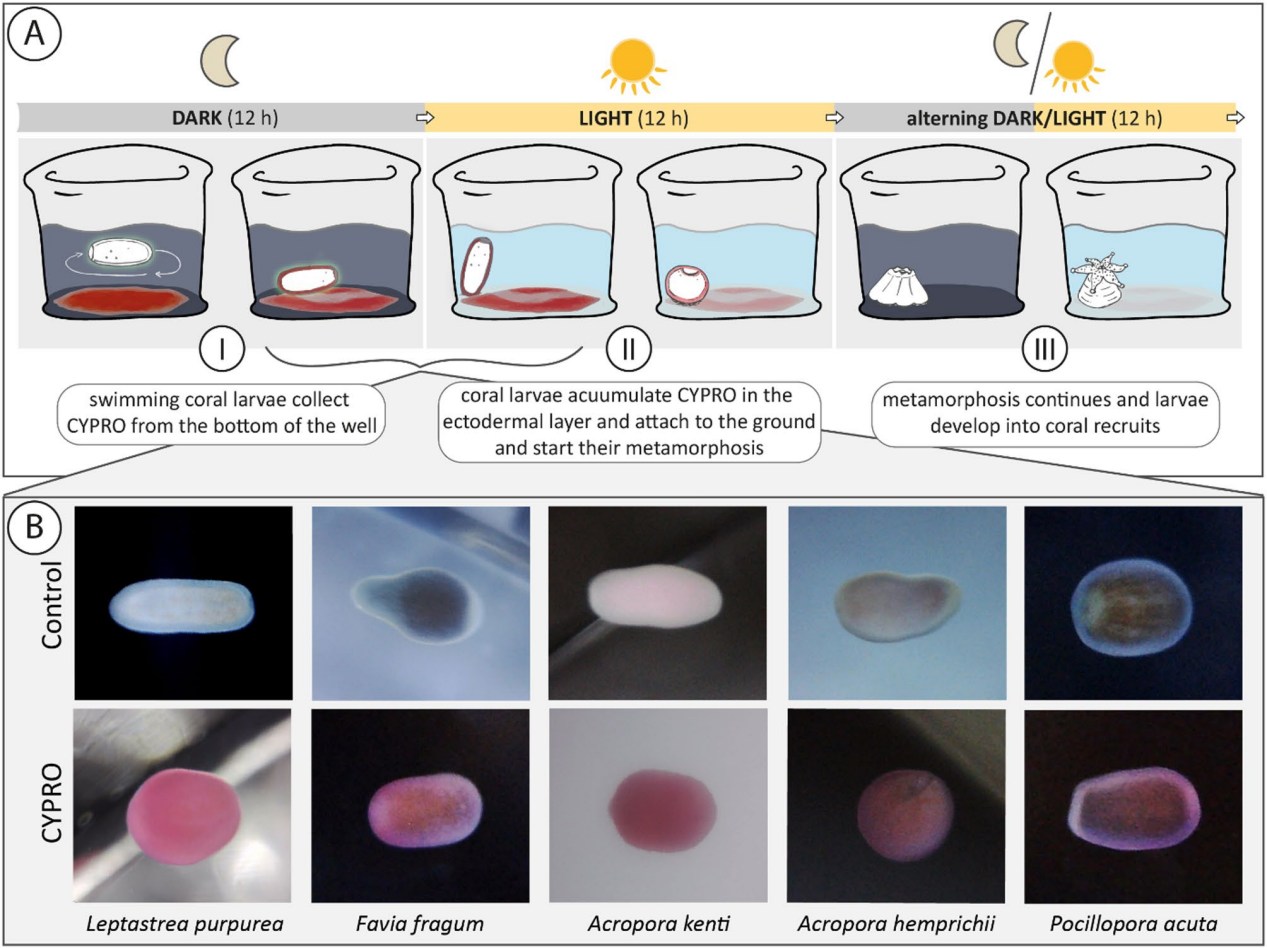
CYPRO uptake and settlement process. (**A**) Conceptual model of settlement with the pigment CYPRO. (A-I) swimming coral larvae accumulate the red pigment CYPRO from the bottom of the well in darkness; (A-II) swimming larvae appear red due to the time-dependent accumulation of CYPRO within their ectodermal layer during the dark period, under light exposure the pigment CYPRO steadily degrades, the strong red color fades over time and with that releases a constant, µM level of H_2_O_2_^50^. Ultimately, the larvae attach to the bottom of the well and proceeds with their metamorphosis into a sessile recruit; (A-III) in the following altering 12 h dark and 12 light phases the pigment CYPRO fades completely, and the larvae further develop into a sessile coral recruits. (**B**) During the initial dark phase of the experiments (first 12 h) the swimming coral larvae of all tested species visibly changed their color and appeared red (cf. CYPRO row). For comparison the pictures at the Control row show the normal coloration of the larvae (without CYPRO).

**Fig. 2:**
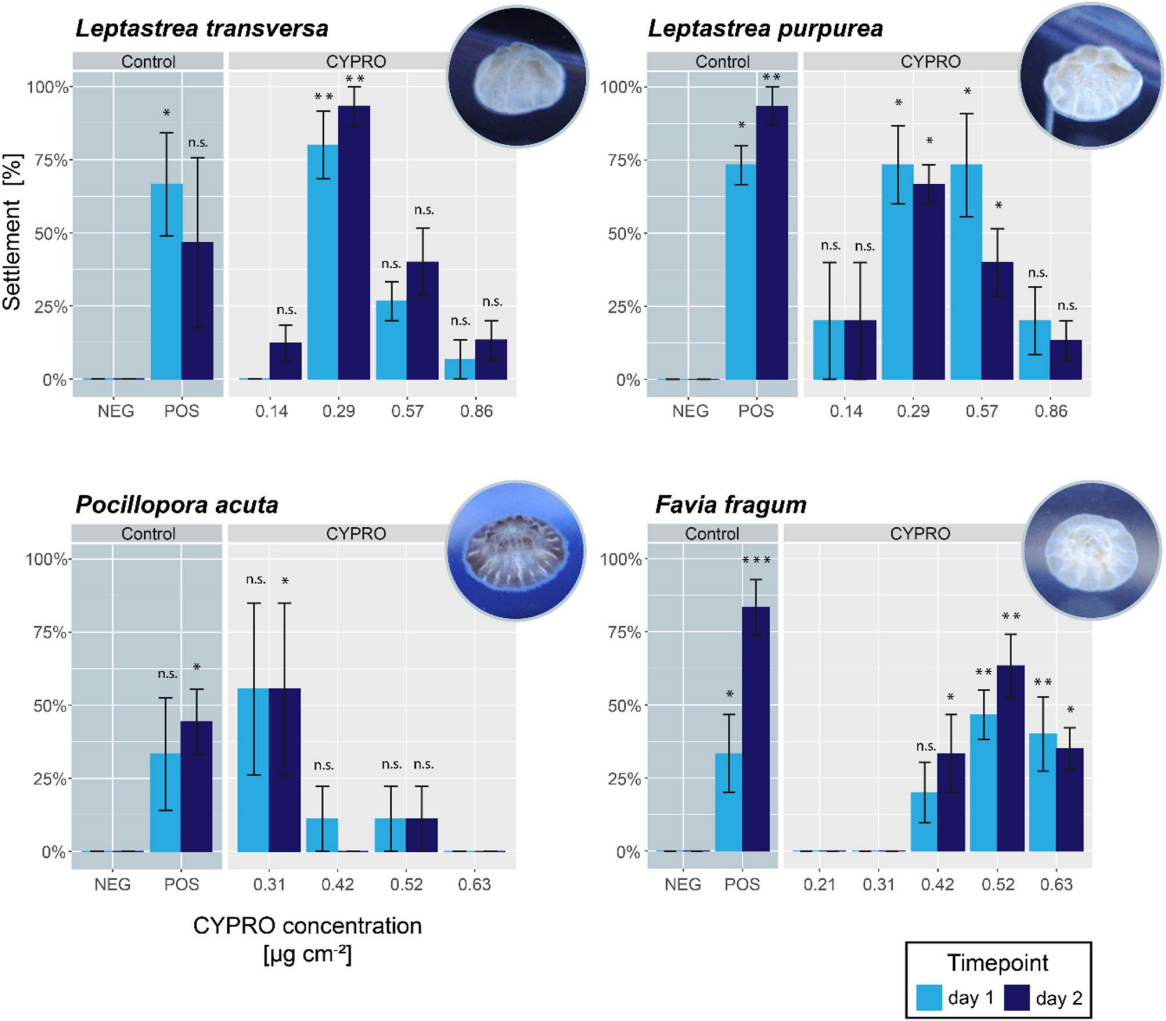
Mean coral larval settlement of brooding species in response to CYPRO. Settlement was monitored after one and two days. Note that CYPRO has a very low water solubility and thus the concentration of the pigment is specified in µg cm^− 2^. Pictures in the upper right corner of each graph show a corresponding coral recruit after 2 days. Error bars represent standard error (SE). Differences within each time point between the negative control and the different CYPRO treatments as well as the positive control were evaluated per experiment using non-parametric Kruskal-Wallis tests, followed by pairwise Dunn tests (α = 0.05). Asterisk indicate the level of significance difference between NEG control and CYPRO treatment or POS control within each timepoint, with n.s. = not significant; * = *p* > 0.05; ** = *p* > 0.005 and *** = *p* > 0.001. *Leptastrea transversa and purpurea*: 3 replicates with 5 larvae each, *Pocillopora acuta*: 3 replicates with 3 larvae each and *Favia fragum*: 6 replicates with 5 larvae each.

**Fig. 3:**
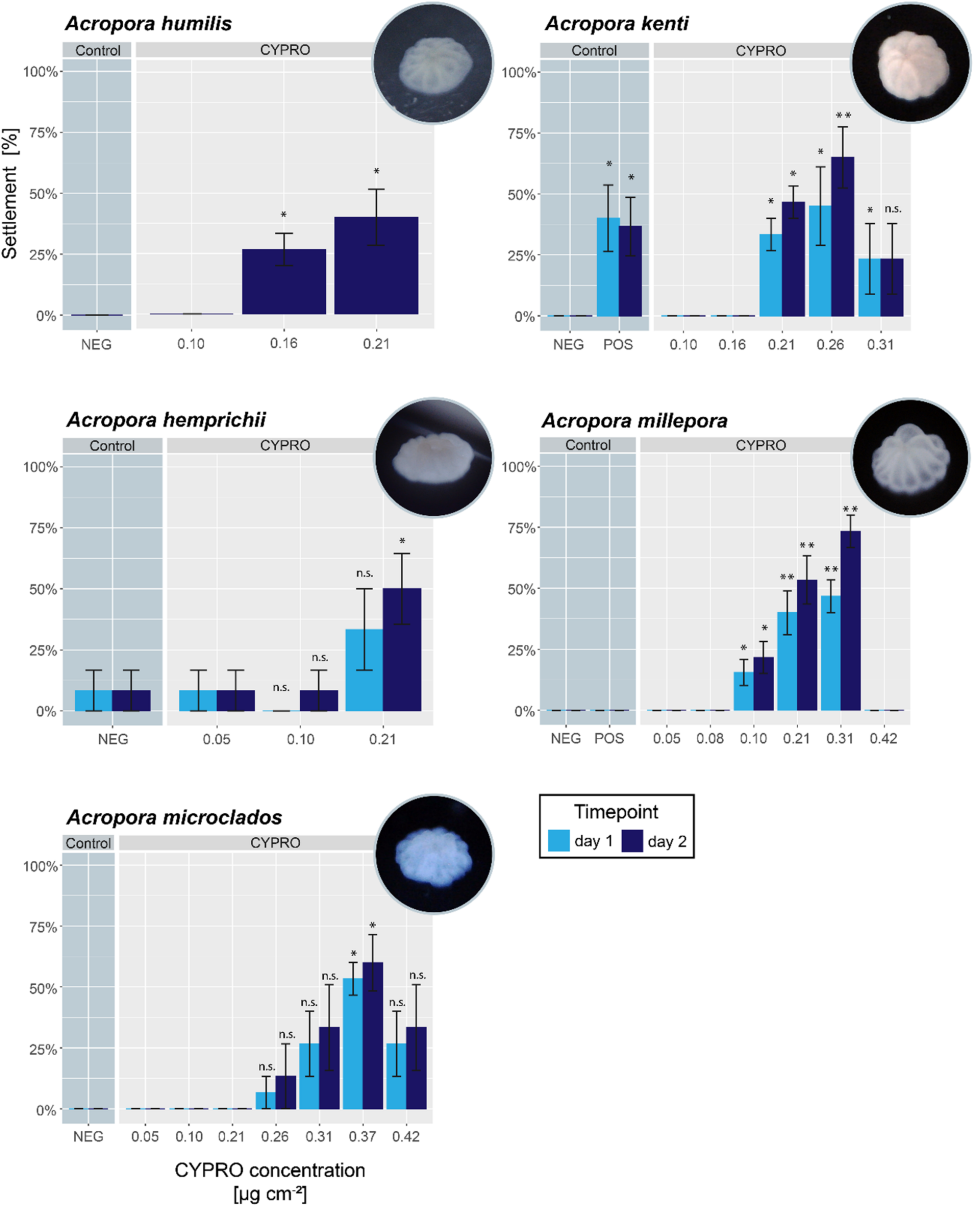
Mean coral larval settlement of broadcast spawning species in response to CYPRO. Settlement was monitored after one and two days. Note that CYPRO has a very low water solubility and thus the concentration of the pigment is specified in µg cm^− 2^. Pictures in the upper right corner of each graph show a corresponding coral recruit after 48 h (day 2). Error bars represent standard error (SE). Differences within each time point between the negative control and the different CYPRO treatments as well as the positive control were evaluated per experiment using non-parametric Kruskal-Wallis tests, followed by pairwise Dunn tests (α = 0.05). Asterisk indicate the level of significance difference between NEG control and CYPRO treatment or POS control within each timepoint, with n.s. = not significant; * = *p* > 0.05; ** = *p* > 0.005 and *** = *p* > 0.001. *Acropora humilis*, *kenti and microclados*: 3 replicates with 5 larvae each, *Acropora hemprichii*: 3 replicates with 4 larvae each (in one control well were 5 instead of 4 larvae),* Acropora millepora*: 3 replicates for the concentrations 0.05; 0.08; 0.31 and 0.42 µg cm^− 2^ and 6 replicates for NEG; POS; 0.1 and 0.21 µg cm^− 2^.

During this study, settlement experiments with nine different coral species (four brooding and five broadcast spawning species) were conducted. In all tested species, the absorption of the red pigment was clearly visible (cf. Figure 1) and ultimately resulted in larval settlement with mean success rates that varied between 40 and 93%, depending on the species and the concentration of CYPRO tested (Figs. 2 and 3). Similarly, metamorphosis in the water column was observed in all species in a concentration dependent manner (cf. Supplemental Figs. 2–10). At the CYPRO concentrations showing the highest settlement success rates, mean water column metamorphosis rates ranged from 7 to 42% and mean mortality rates ranged from 0 to 27%, depending on the species. For each of the nine species (i.e., Figs. 2 and 3), it was possible to identify at least one CYPRO concentration that induced significant settlement (* = *p* > 0.05; ** = *p* > 0.005 and *** = *p* > 0.001) compared to the negative control (NEG) of the same timepoint.

In the brooding species, mean settlement success in the species-specific optimal concentrations varied from 55 to 93% after two days of CYPRO exposure (Fig. 2), while mean metamorphosis in the water column occurred between 7 and 23% (Supplemental Figs. 2–5). *Leptastrea transversa* and *L. purpurea* both had their settlement optimum at a concentration of 0.29 µg cm^− 2^ with mean settlement success at 93 (± 6.7 SE) and 66% (± 6.7 SE), respectively. For *L. purpurea*, 7% of the settled larvae died between the first and the second day. *Pocillopora acuta* larvae settled with 55% (± 29.4 SE) mean success at their optimal CYPRO concentration of 0.31 µg cm^− 2^. *Favia fragum* larvae settled most effectively at a concentration of 0.52 µg cm^− 2^ with a mean success rate of 63% (± 10.9 SE) after two days (Fig. 2) and 73% (±12.3 SE) after 3 days (see Supplemental Fig. 5).

In the spawning species, mean settlement success among the (species-specific) optimal concentrations varied from 40 to 73% after two days of CYPRO exposure (see Fig. 3), while metamorphosis in the water column occurred between 13 and 42% (Supplemental Figs. 6–10). *Acropora humilis* larvae settled most successfully at the highest tested CYPRO concentration of 0.21 µg cm^− 2^, with 40% (± 11.5 SE) mean success rate. *A. kenti* larvae settled best at a CYPRO concentration of 0.26 µg cm^− 2^ with a mean success rate of 60% (± 11.5 SE). *A. hemprichii* larvae settled best when exposed to 0.21 µg cm^− 2^ with 50% mean (± 14.4 SE) success, which was significantly higher compared to NEG, although a small fraction of NEG larvae settled (1 out of 13). This single larva was the only one settling of all 158 NEG control larvae throughout all species. *A. millepora* settled best when treated with 0.31 µg cm^− 2^ with 73% (± 6.7 SE) mean success. Interestingly, larvae of this species did not settle at all in the applied POS control treatment. *A. microclados* settled best when exposed to 0.37 µg cm^− 2^ with 60% (± 11.5 SE) mean success. A detailed description of each data set including all observed larval states such as swimming, attached, metamorphosis in the water column, settlement, deformation and dissolved are provided in the Supplementary section (Supplemental Tables 2 and Supplemental Figs. 2–10).

**Fig. 4:**
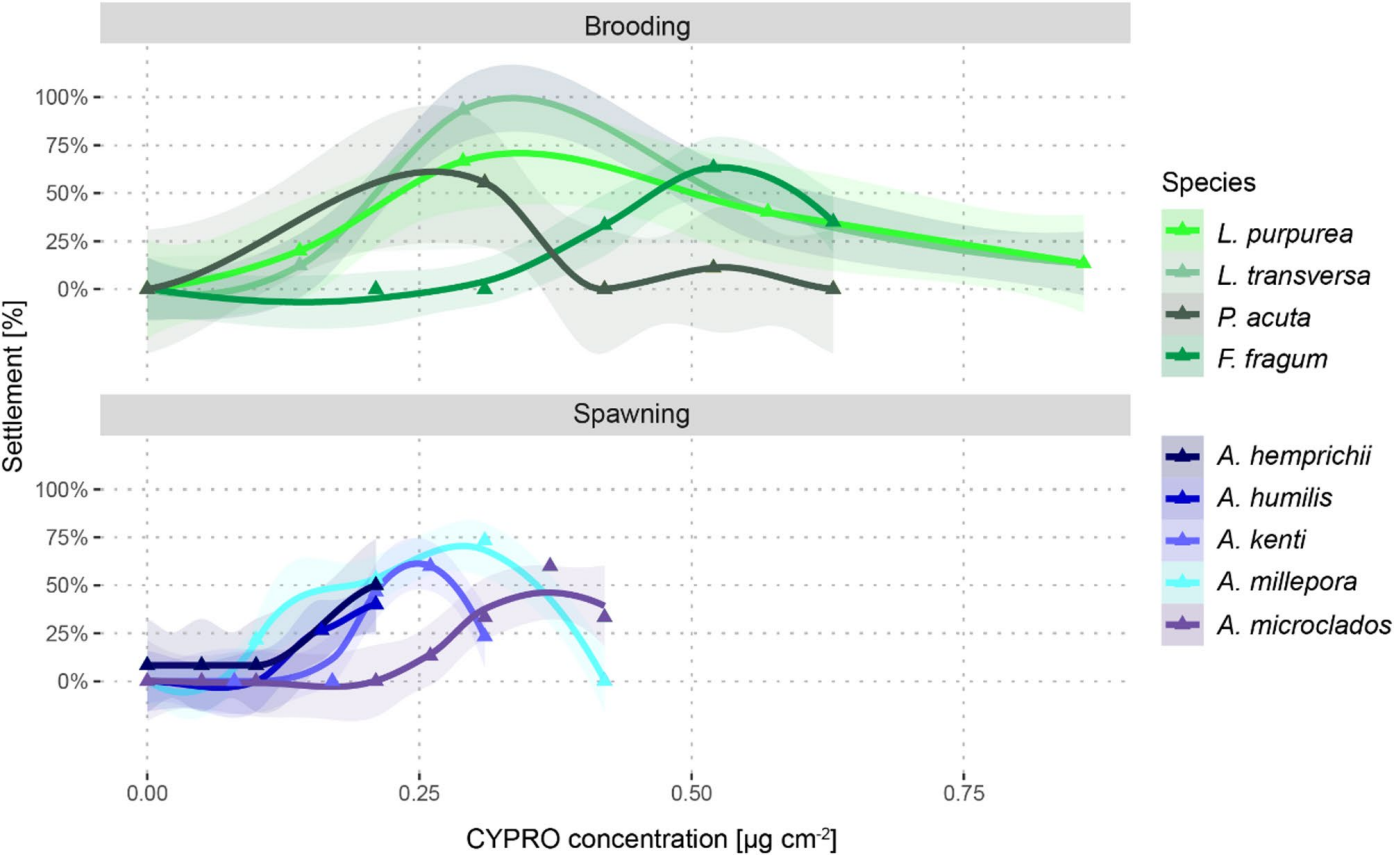
Species-specific settlement-inducing CYPRO concentration ranges separated in brooding and spawning species. Note that for both *Leptastrea* species, smaller well sizes (3.5 cm^2^) were used compared to all other species (9.6 cm^2 ^cf. Table S1). Triangles display the mean settlement in percentage; transparent ribbons display the standard error. Lines in the graph were “loess” smoothed (span = 0.55) using the ggplot2 package.

For the tested coral species, settlement was induced at species-specific optimal CYPRO concentrations (see Fig. 4). For the brooding species, this optimal concentration varied from 0.29 µg cm^− 2^ for both *Leptastrea* species to up to 0.52 µg cm^− 2^ for *F. fragum* larvae. The five spawning *Acropora* species settled most successfully in response to 0.21–0.37 µg cm^− 2^ of CYPRO. For *A. humilis* and *A. hemprichii* the optimal concentration might not have been found, since 0.21 µg cm^− 2^ was the highest tested and most successful settlement concentration.

## Discussion

Coral larval settlement is a crucial process in the life cycle of corals. Yet, it is still not fully understood which biochemical cues are involved and how exactly these cues trigger the settlement reaction of swimming coral larvae. To date, TBP^40^ and CYPRO^47^ are the only identified bacterially produced, multispecies settlement cues for coral larvae. Both compounds were originally isolated from microbial biofilms, or more precisely from the marine bacterial genus *Pseudoalteromonas*. However, recent studies suggest that other bacterial families besides Pseudoalteromonadaceae, not associated with the production of TBP and/or CYPRO, also lead to high settlement rates^24,27,32^. This observation suggests that TBP and CYPRO are likely two of potentially many other settlement cues occurring in natural coral reefs that may function either individually or in combination to induce successful settlement. Nevertheless, the investigation and comparison of purified chemical cues such as TBP and CYPRO offers the opportunity to better understand the settlement induction on a deeper mechanistical level. Both compounds were observed to induce to some extent metamorphosis in the water column (without prior attachment), if applied in a non-optimal concentration range (cf. Supplemental Figs. 2–10^38,40,41^). Furthermore, both compounds are associated to the production of reactive oxygen species such as H_2_O_2_^49,50^ which, if solely applied, induces metamorphosis in the water column^50,51^. Petersen and colleagues^50^ hypothesized that the role of H_2_O_2_ released during CYPRO degradation is one of the main drivers of this chemically induced settlement mechanism. Using light and confocal microscopy, the latter authors observed that larvae of the brooding species *L. purpurea* were able to absorb CYPRO into their outer ectodermal layer, clearly changing their appearance from a translucent-brownish color to a red coral larva after a few hours of CYPRO exposure. This study extends the use of CYPRO as settlement cue to four brooding and four broadcast spawning coral species and found that all eight additionally tested species rapidly took up CYPRO, which likewise resulted in a clearly visible red coloration (cf. Figure 1B). However, this “blushing” of the larvae only occurred in complete darkness and when exposed to rather high CYPRO concentrations. The addition of CYPRO under direct exposure to light always led to a rapid and visible decrease of the pigment at the bottom of the well, and the larvae retained its translucent-brownish color. Thus, this study supports the generality of the conceptual model of a CYPRO-induced coral settlement mechanism (time-dependent accumulation of CYPRO within the larvae during darkness followed by photodegradation of CYPRO during daylight and with that release of µM H_2_O_2_ levels, cf. Figure 1A) proposed by Petersen and colleagues^50^. Given the high settlement rates of CYPRO in all nine brooding and broadcast spawning coral species, we propose CYPRO as a general settlement cue for scleractinian corals, although future research should identify species-specific optimal concentrations of CYPRO to maximize settlement success and possible applications in coral restoration.

Factors such as larval size and their swimming behavior might influence the uptake rate of the chemical cue by the searching larvae. For example, larger larvae with a greater surface area need to accumulate more of the lipophilic cue. In addition, larvae that search horizontally (i.e., *L. purpurea*) appear to collect more of the pigment within a given time interval than larvae that mainly move vertically in the water column, such as observed in the five tested *Acropora* species. Thus, the offered CYPRO concentrations may have been too low to trigger maximum settlement rates for some tested species. For example, *A. hemprichii* and *A. humilis* achieved highest settlement rates with the highest tested CYPRO concentration of 0.21

µg cm^− 2^ (cf. Figure 3). The species’ optimal concentration might be higher than 0.21 µg cm^− 2^, although this could not be tested due to a lack of larval supply. *P. acuta*, on the other hand, settled with highest success rates at the lowest applied CYPRO concentration of 0.31 µg cm^− 2^ and might settle with higher success when exposed to lower CYPRO concentrations (< 0.31 µg cm^− 2^, cf. Figure 2). If too high CYPRO concentrations were applied, it resulted in the mortality of the larvae (cf. Supplemental Figs. 2–10). Other factors influencing success in settlement may be caused by differences in uniform illumination of the experimental set ups or in the thickness and surface distribution of the applied CYPRO film at the bottom of the well. For example, if CYPRO is applied in a 10-fold higher concentrated MeOH solution (e.g. 1 mg mL^− 1^), the distribution of the dried pigment is less homogenous, and the searching larvae may take up either too much or too little of CYPRO depending on the area that the individual larva has swept. All these aspects may lead to variations in settlement rates among individual larvae and coral species and should be considered to optimize settlement success. In some experiments, only low amounts of larvae were available, resulting in a low number of replicates, especially in the case of *P. acuta*, which was tested using three replicates per treatment with three larvae each, but nevertheless showed significant results compared to the NEG control. Even though the experimental design could be improved, this study clearly shows that CYPRO is a multispecies settlement cue and thus holds a great potential for broad usage.

In coral restoration based on larval recruitment, more attention has recently been directed towards chemically induced settlement^7,10^ as it appears to be more reliable, better available and easier to manage than biological cues (e.g., CCA, biofilms). This approach currently utilizes purified chemical settlement inducers such as TBP or Hym-248, that are both commercially available (TBP - Aaron Chemicals LLC, USA) and (Hym-248 - NovoPro, USA). However, aside from their varying efficiency and the species specificity of Hym-248 ^36,38–44^, both chemical cues are highly water soluble, making their use in large aquarium systems or open ocean reef environments extremely challenging. Furthermore, the chemical properties of TBP such as its instability in storage and transport to remote areas^39^ as well as the elevated toxicity for phytoplankton^52^ may be problematic for large-scale reef restoration programs. CYPRO (available at Sirius Fine Chemicals SiChem GmbH, Germany), is suitable for long term storage (> 2 years at -20 °C) and prolonged transport at room temperatures if stored in complete darkness. Furthermore, CYPRO strong lipophilicity allows its application on substrates such as settlement tiles, regardless of the water volume. Its use on non-conditioned, biofilm-free tiles might have positive effects on coral post-settlement survival. Specifically, it could provide more time for corals to reach a size-escape threshold before tiles are colonized by strong benthic competitors, such as macroalgae and bryozoans.

In conclusion, we showed that CYPRO is functioning as a multispecies settlement cue for a wide range of brooding and broadcast spawning scleractinian coral species. Since an increased effectiveness of restoration efforts in the early life-stages holds the potential to significantly improve the restoration outcome, CYPRO, is an interesting settlement cue for large-scale coral reef restoration. Future studies should use CYPRO as a tool for reef restoration, focusing on settlement efficiency and post-settlement survival rates.

## Materials and methods

### Larval acquisition

Coral larvae of the brooding species *Leptastrea purpurea* and *Leptastrea transversa* were collected in the facility of the Marine Institute in Guam (USA) and larvae of the species *Pocillopora acuta* were collected in the aquarium facility of the Environmental Chemistry Group of the University of Oldenburg in Wilhelmshaven (GER). For these three species, a similar collection procedure was used^53^. In brief, parental colonies were placed in a potassium chloride (KCl) bath (5–7.5 g L^− 1^) for a period of 5 to 10 min after which they released fully developed and competent swimming larvae. Larvae were immediately collected using a plastic Pasteur pipette and washed at least two times by transferring them into a bath of filtered (using a 0.2 μm nylon filter) natural sea water for both *Leptastrea* species or artificial sea water (FASW, Tropic Marin^®^ Pro-Reef Salt, Wartenberg, Germany) for *Pocillopora acuta*. Brooded larvae were stored for a maximum of 3 weeks in an incubator (Lovibond) at a steady temperature of 26 °C and a 12 h dark/light rhythm. The FASW media was exchanged weekly. *Favia fragum* larvae were collected at the aquarium facility of the University of Wageningen (NL) following the collection procedure described in^54^. In brief, adult colonies were placed individually in 1 L beakers during the night. The beaker had a constant inflow of 27 °C FASW that continuously caused buoyant larvae to overflow over the handle into a collection container. Larvae were transferred into a storage container filled with 500 mL of 0.5 μm FASW.

Spawned larvae were obtained within the artificial spawning system of the Environmental Chemistry Group of the University of Oldenburg in Wilhelmshaven (GER), based on the technology of the Coral Spawning Lab Ltd (GB). The underlying principle of these systems is to induce bundle generation and synchronized spawning in parental coral colonies by imitating the lunar cycle as well as seasonal variations in the natural day length and water temperature^55^. As soon as the setting (i.e., appearance of sperm-egg bundles) of the parental colonies was visible, a tube of acrylic glass was placed around the colonies to capture ascending sperm-egg bundles. After bundle collection and their disintegration, sperm and eggs were separated with a 100 μm mesh. Sperm fractions of all spawned colonies were combined and used to fertilize the eggs of all suitable colonies to increase potential genetic diversity. Fertilized eggs further developed into swimming coral larvae in circular mesh-bottom containers floating in a larger holding tank of a recirculating aquaculture system with a steady but slow water movement. The layers of fat accumulating on the water surface from remaining sperm and dissolving eggs were removed periodically using pieces of plastic wrap to ensure sufficient water quality. Depending on the coral species, larvae gained competence to settle within 5 to 7 days after fertilization. For more specific information see Table S1.

### CYPRO production

CYPRO was acquired using the cultivation and extraction protocol as described by^47^ but is also commercially available at Sirius Fine Chemicals SiChem GmbH. In brief, Marine Broth (MB, Carl Roth GmbH) agar plates were inoculated with a *Pseudoalteromonas rubra* cryo-conserved stock that was stored at -80 °C. After 48 h, an individual colony was picked and transferred into MB liquid media. After another 24 h, the yellow-transparent liquid media changed into a milky-cloudy state. In that state, the liquid media was used to inoculate MB agar plates, which were incubated at room temperature and in darkness for another 24 h. The grown bacterial lawn was scraped off and extracted in methanol (MeOH) first by homogenization using an immersion blender (Ultra-Turrax T25, Janke & Kunkel IKA Labortechnik) at 12,000 rpm for 30 s followed by a 10 s ultrasonic bath (Bandelin Sonorex). The extract was then filtered through cellulose filter paper (grade 3 hw, sartorius) and dried with a speed-vac system (Christ, Gefriertrocknungsanlagen GmbH). The dried crude extract was re-dissolved in MeOH and further purified using a combined approach of liquid-liquid partitioning (LLP), a C_18_-based solid-phase extraction (SPE, SUPELCO) and high-performance liquid chromatography with a semi-preparative C_18_-column and for peak-base separation a diode array detector (HPLC-DAD, Agilent Technologies). The quality of the separation process was tested subsequently, using ultrahigh resolution mass spectrometry^47^. All production steps were performed in low-light environments to prevent a light-induced breakdown of CYPRO. Purified CYPRO was stable for at least 2 years when stored in the dark either dry or dissolved in MeOH at -20 °C. The stored CYPRO amount was quantified by HPLC-MS and additionally analyzed for degradation products.

### Settlement experiments

**Fig. 5:**
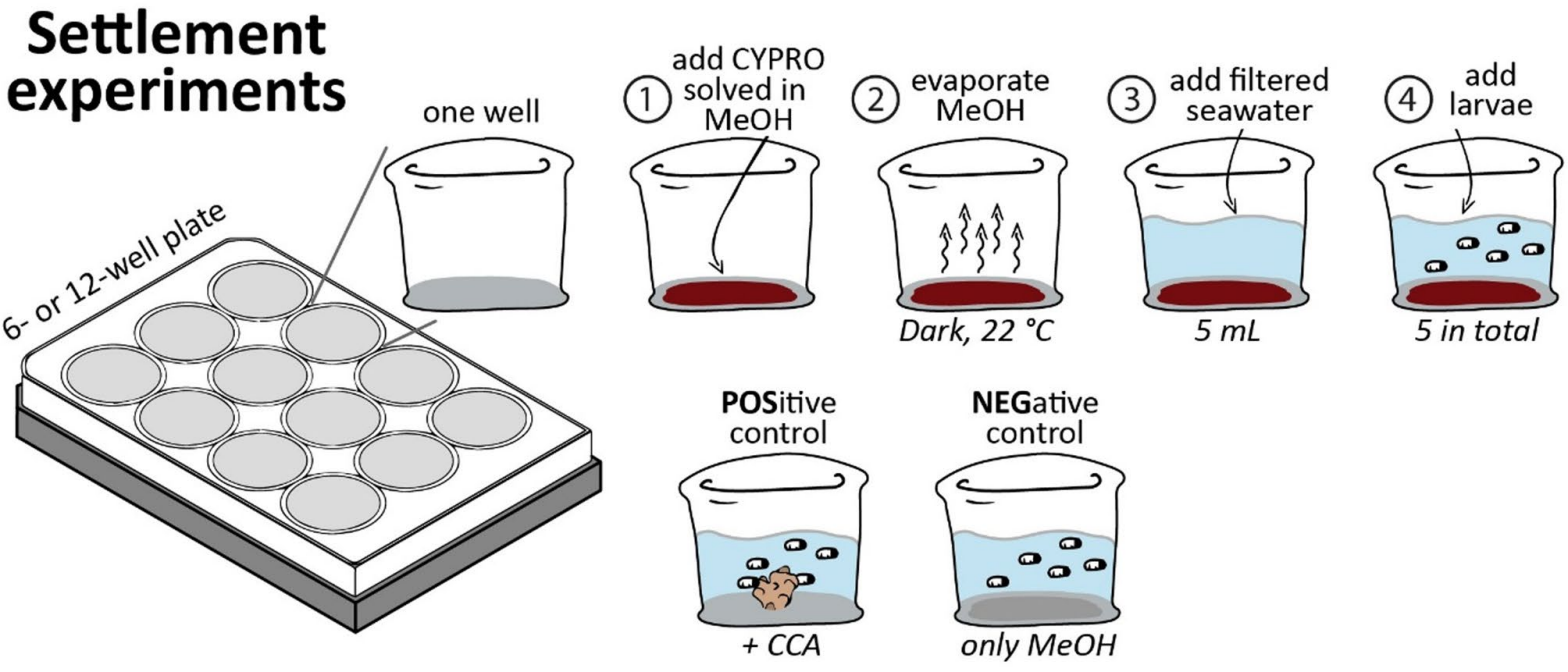
Stepwise instructions to the conducted settlement experiments. (1) Addition of CYPRO dissolved in MeOH, (2) evaporation of MeOH with a remaining CYPRO film on the bottom, (3) addition of FASW and (4) addition of larvae. For specific information about each experiment see Figure S1. CYPRO has an extremely high lipophilicity and therefore does not dissolve in the FASW (step 3). Since it remains on the bottom of the plate, CYPRO concentrations are provided in µg cm^− 2^.

To obtain high settlement rates with each coral larval species, CYPRO was tested at a range of concentrations (i.e. 0.05–0.86 µg cm^− 2^). Settlement experiments were conducted in 6-well (9.6 cm^2^) or 12-well (3.5 cm^2^) cell culture plates using a similar protocol as described in^48^ (cf. Figure 5). The purified settlement cue CYPRO was dissolved in MeOH to a concentration of 0.1 mg mL^− 1^ and the tested quantities were pipetted on the bottom of the well plate. The organic solvent MeOH was evaporated at room temperature for approximately 30 min in the absence of light to prevent degradation of the pigment CYPRO. Subsequently, a thin CYPRO film remained on the bottom of the well, however not homogenously distributed over the entire well surface. As negative control (NEG), only MeOH without CYPRO was pipetted onto the bottom of the well, using the corresponding maximum volume used in the assays with CYPRO. A positive control (POS) was used in experiments in which sufficient larvae were available (see Table S1). For that, an approximately 1 cm^2^ piece of live CCA either cultured in the aquarium facilities in Wilhelmshaven or freshly collected CCA pieces of the species *Hydrolithon reinboldii* from the Luminao Reef in Guam (USA) was used (cf. Table S1). Finally, the wells were filled either with 5 mL (12-well plate) or 10 mL (6-well plate) of FASW and 3 or 5 larvae were added to each well (cf. Table S1). Since CYPRO has an extremely high lipophilicity it hardly dissolves in the FASW and remains on the bottom of the well plate. All laboratory experiments were incubated constantly at 26 °C and began with a 12 h dark phase followed by 12 h of light exposure (65 µmol m^− 2^ s^− 1^; see Supplemental Fig. 1 for detailed light spectra) alternating until the end of the experiment. Experiments with both *Leptastrea* species were conducted in the outdoor facilities of the University of Guam Marine Laboratory at an average temperature of 28 °C and started with 8 h darkness followed by the natural light rhythm and composition. Settlement was determined as a change from the free-swimming, pear-shaped larval form to a disc-shaped recruit, that was attached solidly to the well, with septal mesenteries radiating from the mouth as defined by Heyward and Negri^56^. The experiments were monitored after approx. 24 h and 48 h. Larvae were tested for adhesion after two days with a gentle flow of water induced by a pipette. For detailed specifications of each experiment see Table S1.

### Statistical analysis

In each experiment at least three replicates (wells) per control and treatment with at least three larvae per well were tested for settlement (for detailed information about each experiment see Table S1). Differences in settlement percentage between the negative control and the different CYPRO treatments as well as the positive control were evaluated per treatment and within timepoint (day 1, day 2) using non-parametric Kruskal-Wallis tests, followed by pairwise Dunn tests (α = 0.05) without corrections for multiple comparisons. R (version 4.3.2) with the RStudio IDE (version 2023 9.1.494) was used for data analysis. The packages dunn.test 1.3.5 ^57^ and tidyverse 2.0.0 ^58^ were used for statistical testing, data manipulation and plotting.

## Electronic supplementary material

Below is the link to the electronic supplementary material.

Supplementary Material 1

Supplementary Material 2

## Data Availability

All data generated or analyzed during this study are included in this published article or in the Supplemental Materials.
