# Supplementary material for: Cycloprodigiosin: A multispecies settlement cue for scleractinian coral larvae
Source: Sci Rep. 2025 Jul 25;15:27075. doi: 10.1038/s41598-025-12409-5 (PMC12297242; doi:10.1038/s41598-025-12409-5)
Supplement: Supplementary file 2 — Supplementary Material 2 [file 41598_2025_12409_MOESM2_ESM.docx]

**Supporting Information for**

**Cycloprodigiosin: A multispecies settlement cue for scleractinian coral larvae**

Laura J. Fiegel*, Samuel Nietzer, David Brefeld, Robbert C. Geertsma, Ronald Osinga,
Peter J. Schupp* and Matthias Y. Kellermann

*** Corresponding Authors:**

Laura J. Fiegel,

Address: Institute for Chemistry and Biology of the Marine Environment (ICBM), Carl-von-Ossietzky University Oldenburg, Schleusenstrasse 1, 26382 Wilhelmshaven, Germany,

Phone Number: +49 4421 944 106
E-Mail: Laura.fiegel@uni-oldenburg.de

Peter J. Schupp,

Address: Institute for Chemistry and Biology of the Marine Environment (ICBM), Carl-von-Ossietzky University Oldenburg, Schleusenstrasse 1, 26382 Wilhelmshaven, Germany,

Phone Number: +49 4421 944 100

E-Mail: Peter.schupp@uni-oldenburg.de

**This PDF file includes:**

Figures S1 to S10

Tables S1 to S2

SI References


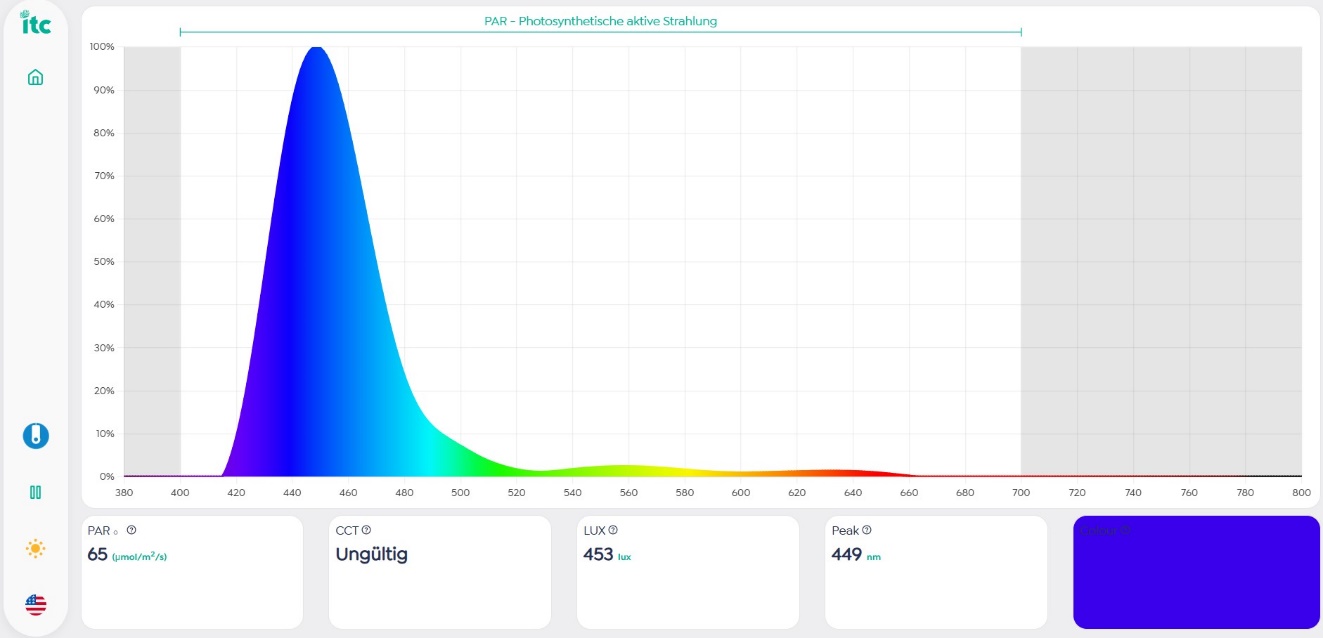


Supplemental Figure 1. Artificial light composition and intensity applied in all experiments except for both *Leptastrea* species (*cf.* Sup. Table 1) using Radion G6 XR15 and XR30 lamps (EcoTech Marine).


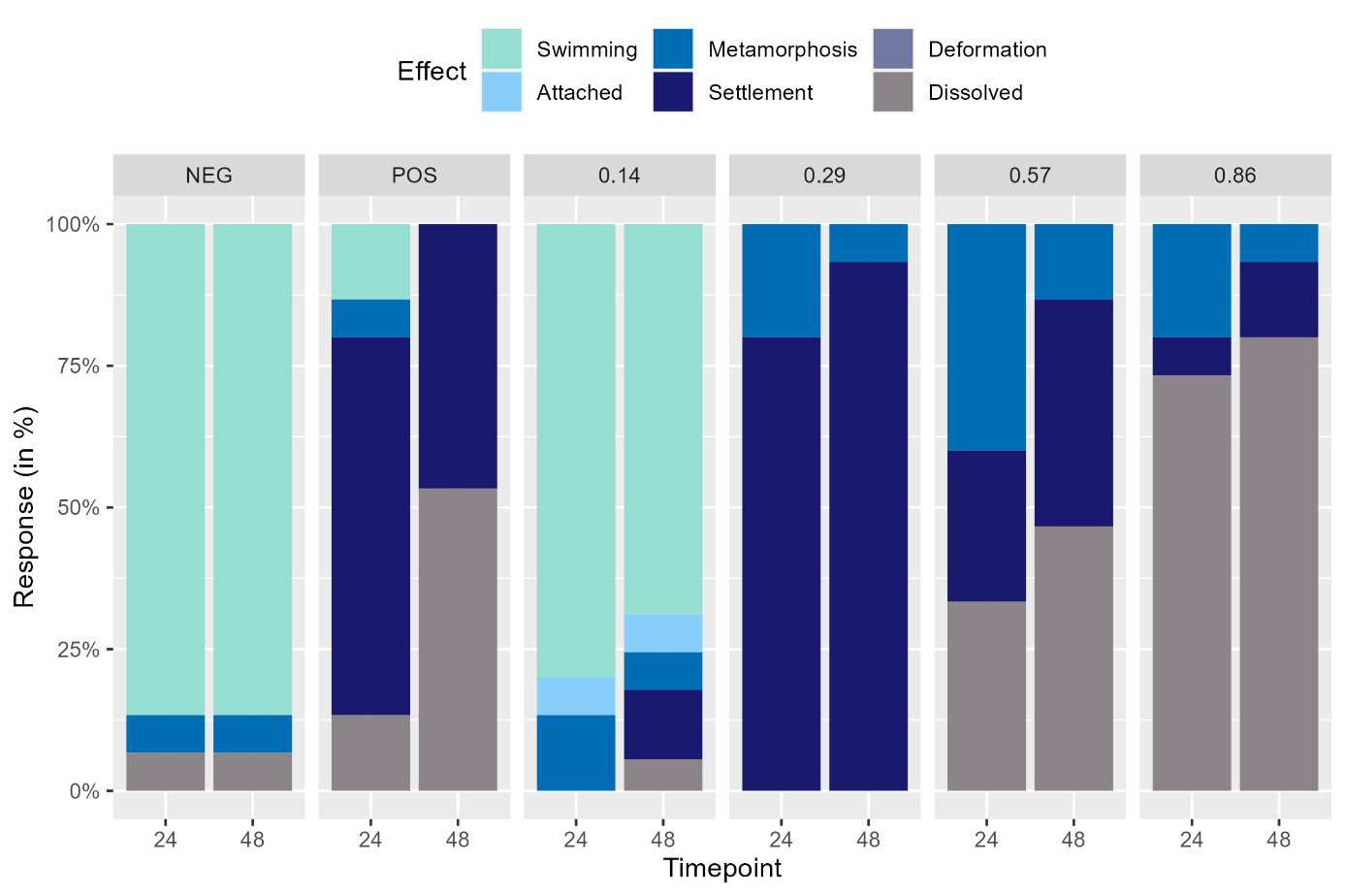


Supplemental Figure 2. Detailed demonstration of the mean effects of CYPRO in different concentrations (0.14, 0.29, 0.57 and 0.86 µg cm^-2^) on *Leptastrea transversa* larvae after 24 and 48 h using 3 replicates with 5 larvae each.


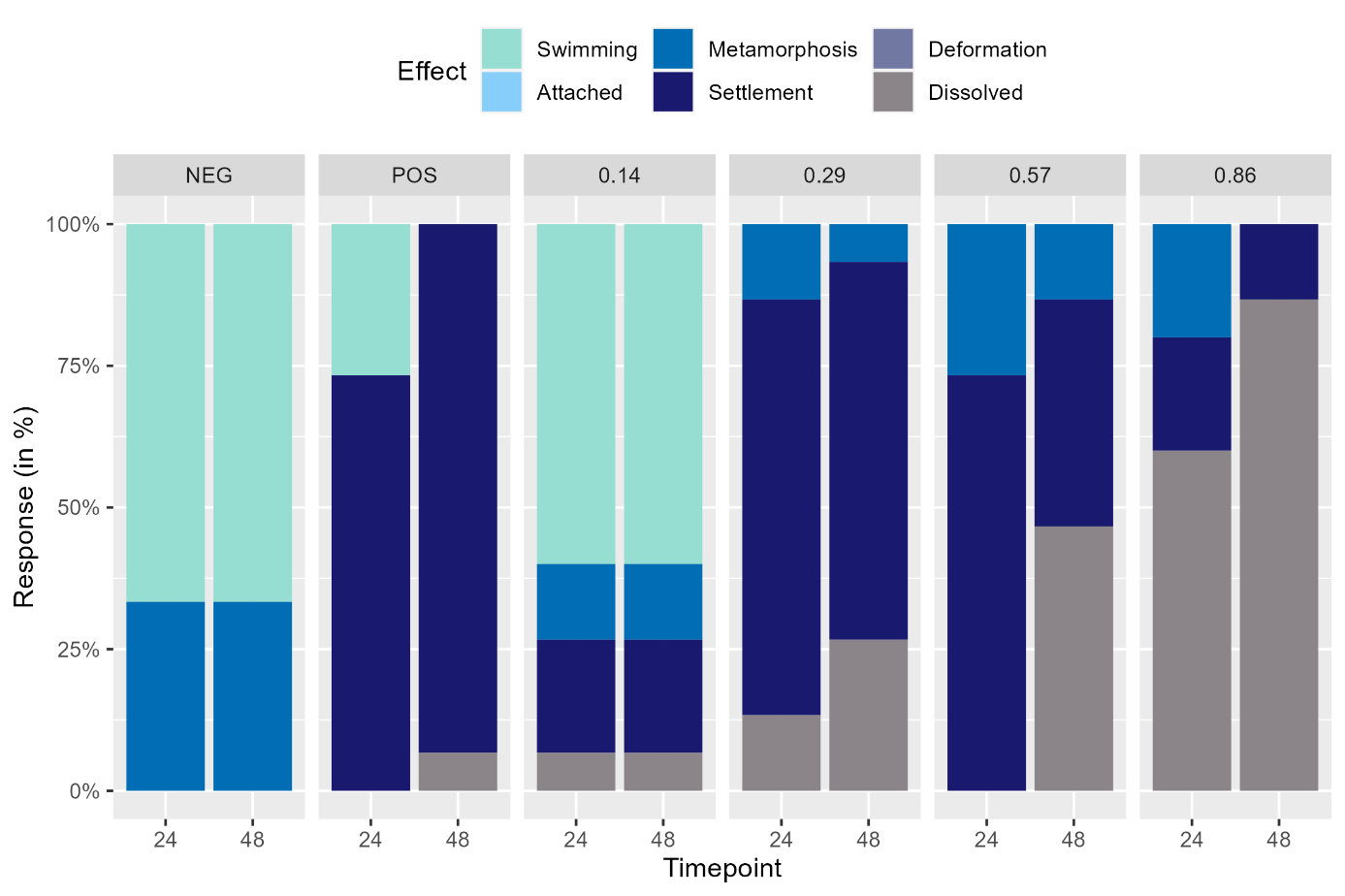


Supplemental Figure 3. Detailed demonstration of the mean effects of CYPRO in different concentrations (0.14, 0.29, 0.57 and 0.86 µg cm^-2^) on *Leptastrea purpurea* larvae after 24 and 48 h using 3 replicates with 5 larvae each.


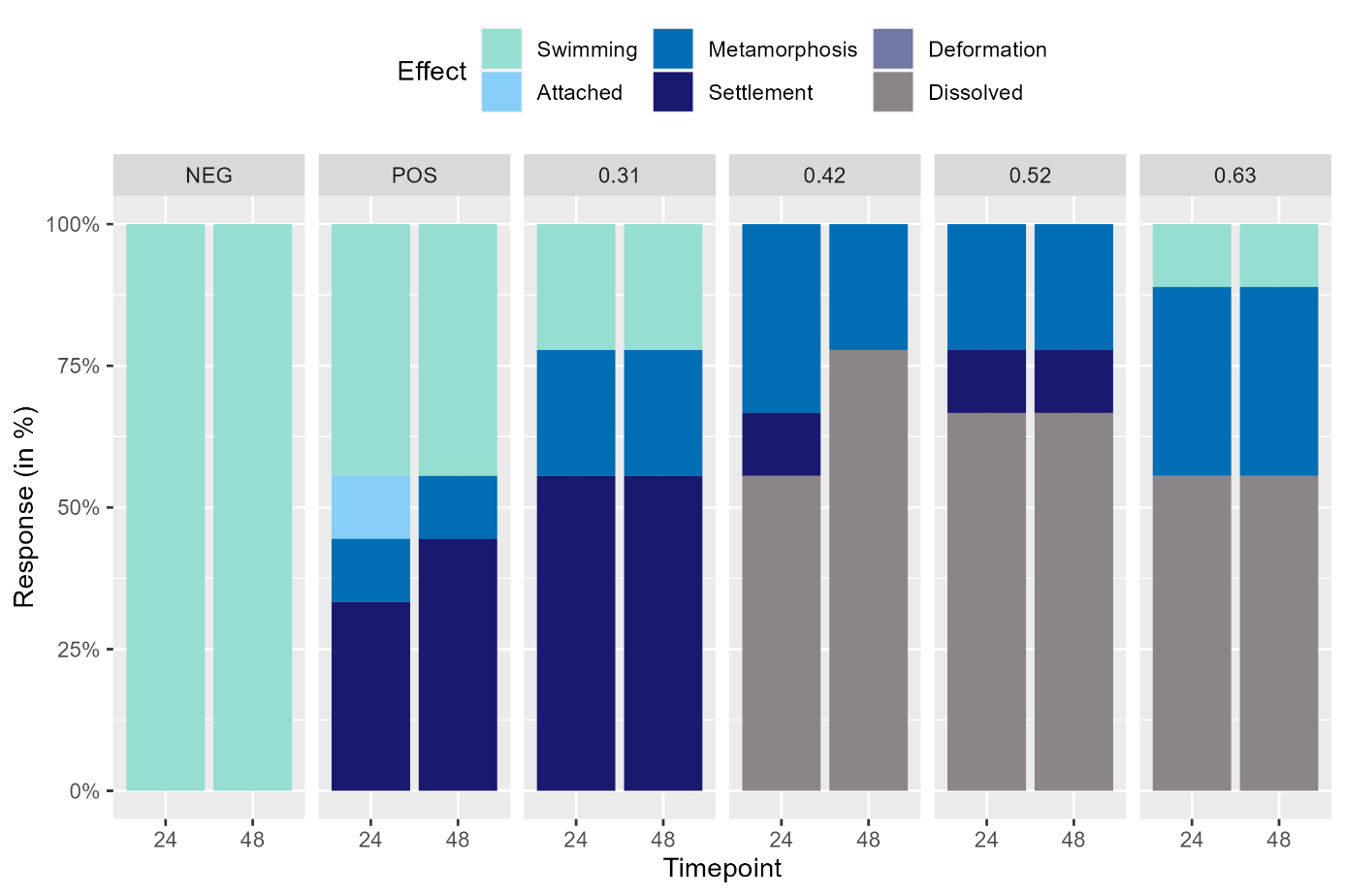


Supplemental Figure 4. Detailed demonstration of the mean effects of CYPRO in different concentrations (0.31, 0.42, 0.52 and 0.63 µg cm^-2^) on *Pocillopora acuta* larvae after 24 and 48 h using 3 replicates with 3 larvae each.

**
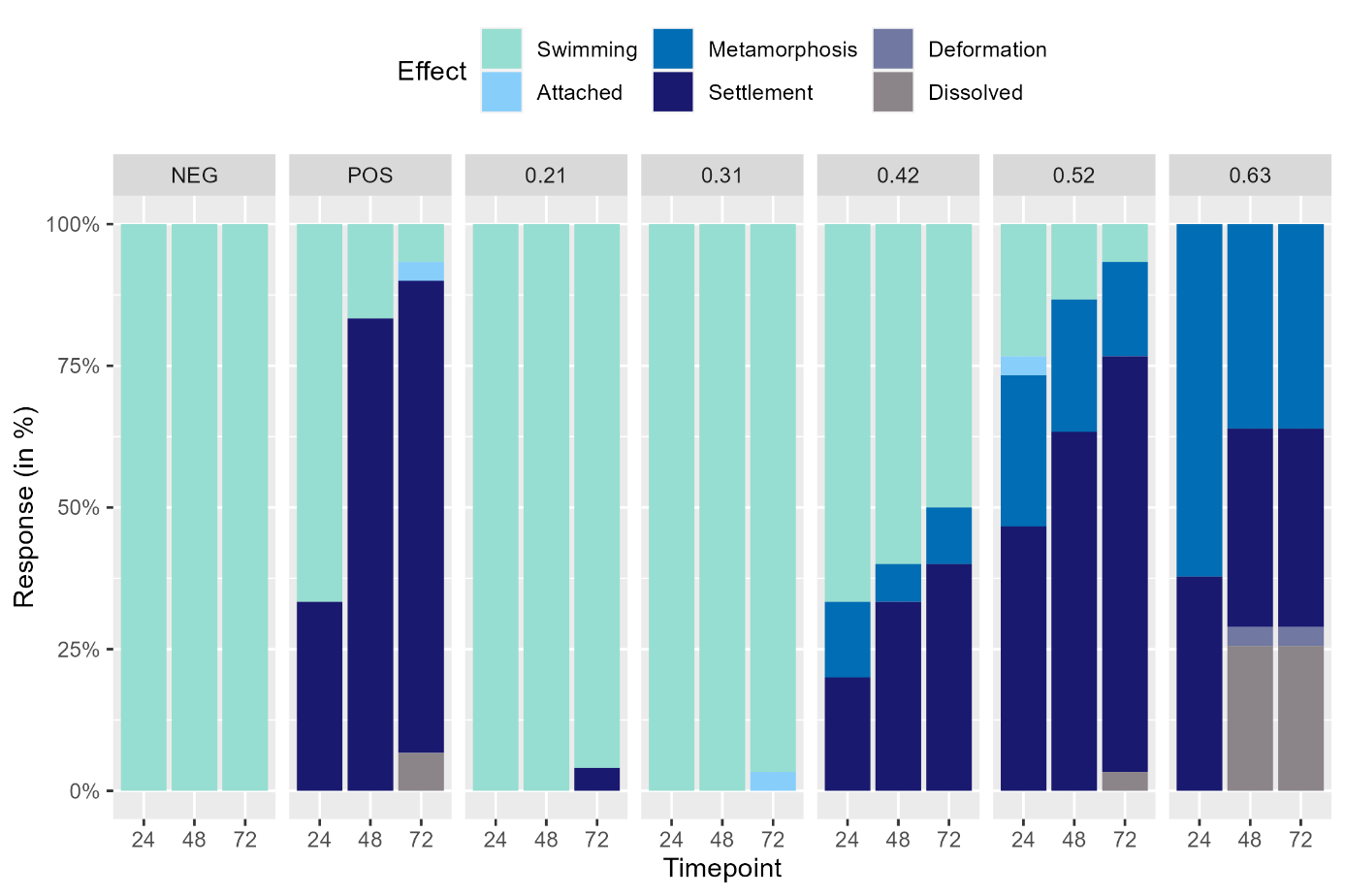
Supplemental Figure 5.** Detailed demonstration of the mean effects of CYPRO in different concentrations (0.21, 0.31, 0.42, 0.52 and 0.63 µg cm^-2^) on *Favia fragum* larvae after 24 and 48 and 72 h using 6 replicates with 5 larvae each.


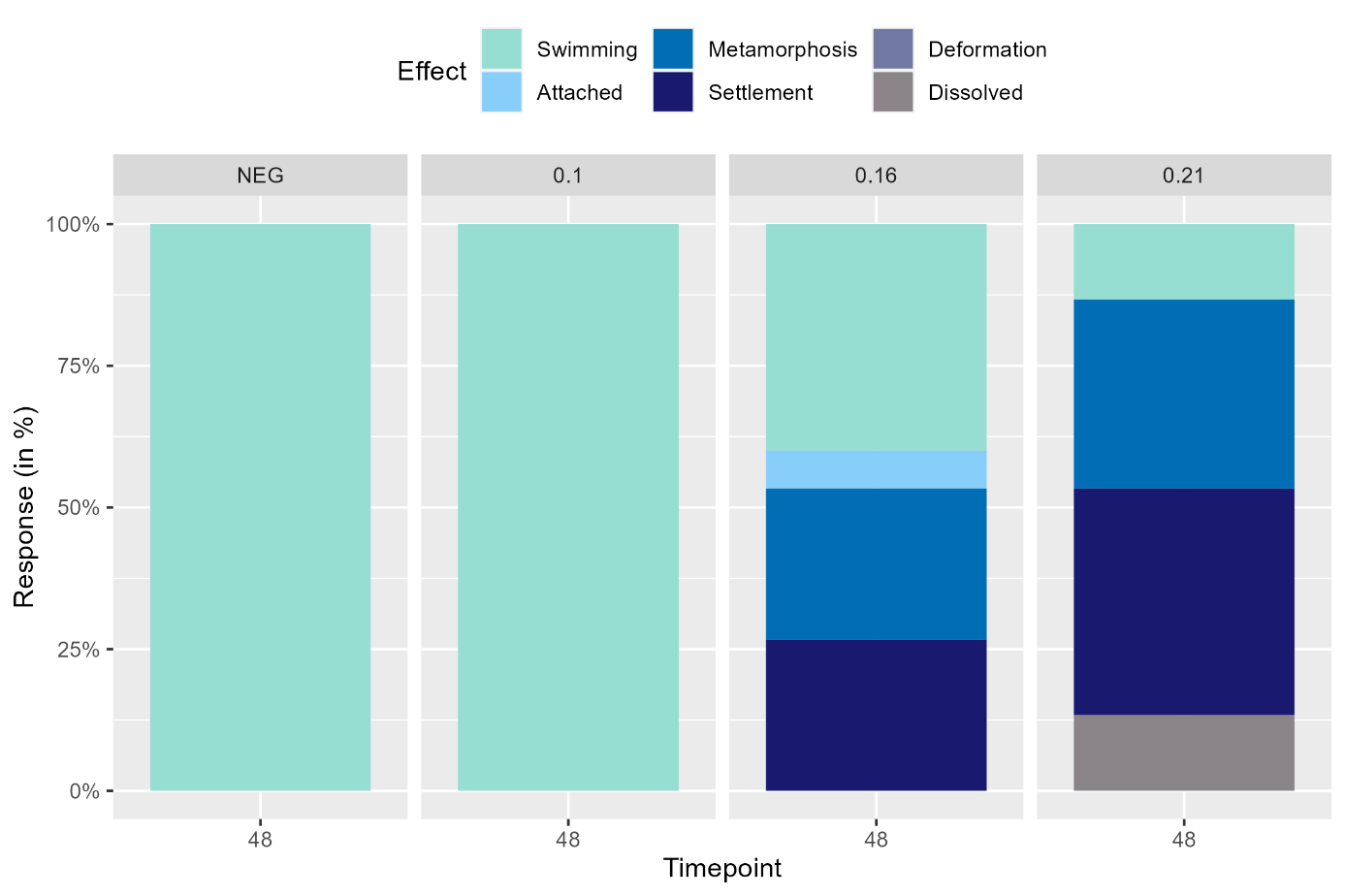


Supplemental Figure 6. Detailed demonstration of the mean effects of CYPRO in different concentrations (0.1, 0.16 and 0.21 µg cm^-2^) on *Acropora humilis* larvae after 48 h using 3 replicates with 5 larvae each.


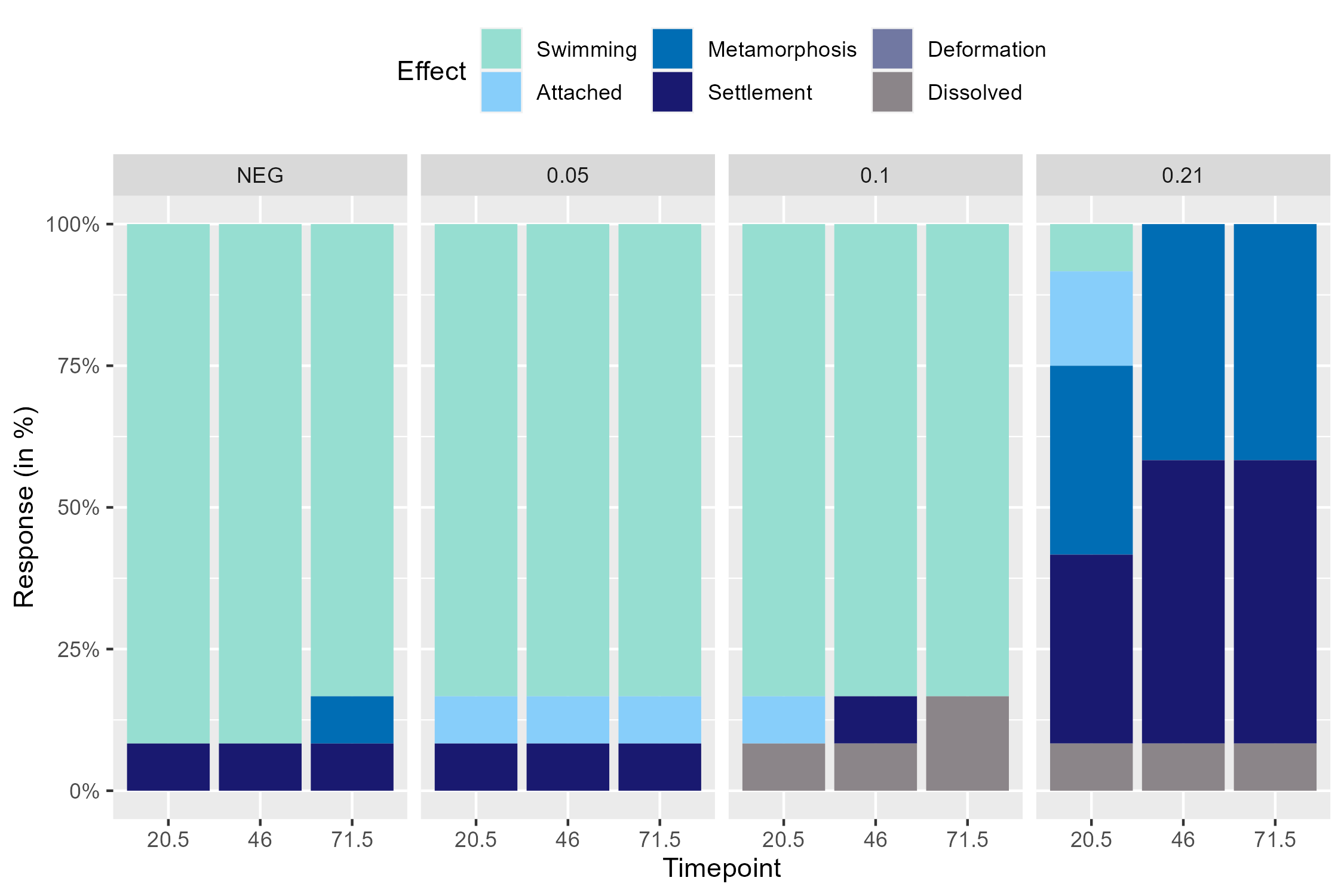


Supplemental Figure 7. Detailed demonstration of the mean effects of CYPRO in different concentrations (0.05, 0.1 and 0.21 µg cm^-2^) on *Acropora hemprichii* larvae after 20.5, 46 and 71.5 h using 3 replicates with 4 larvae each (in one control well were 5 instead of 4 larvae).


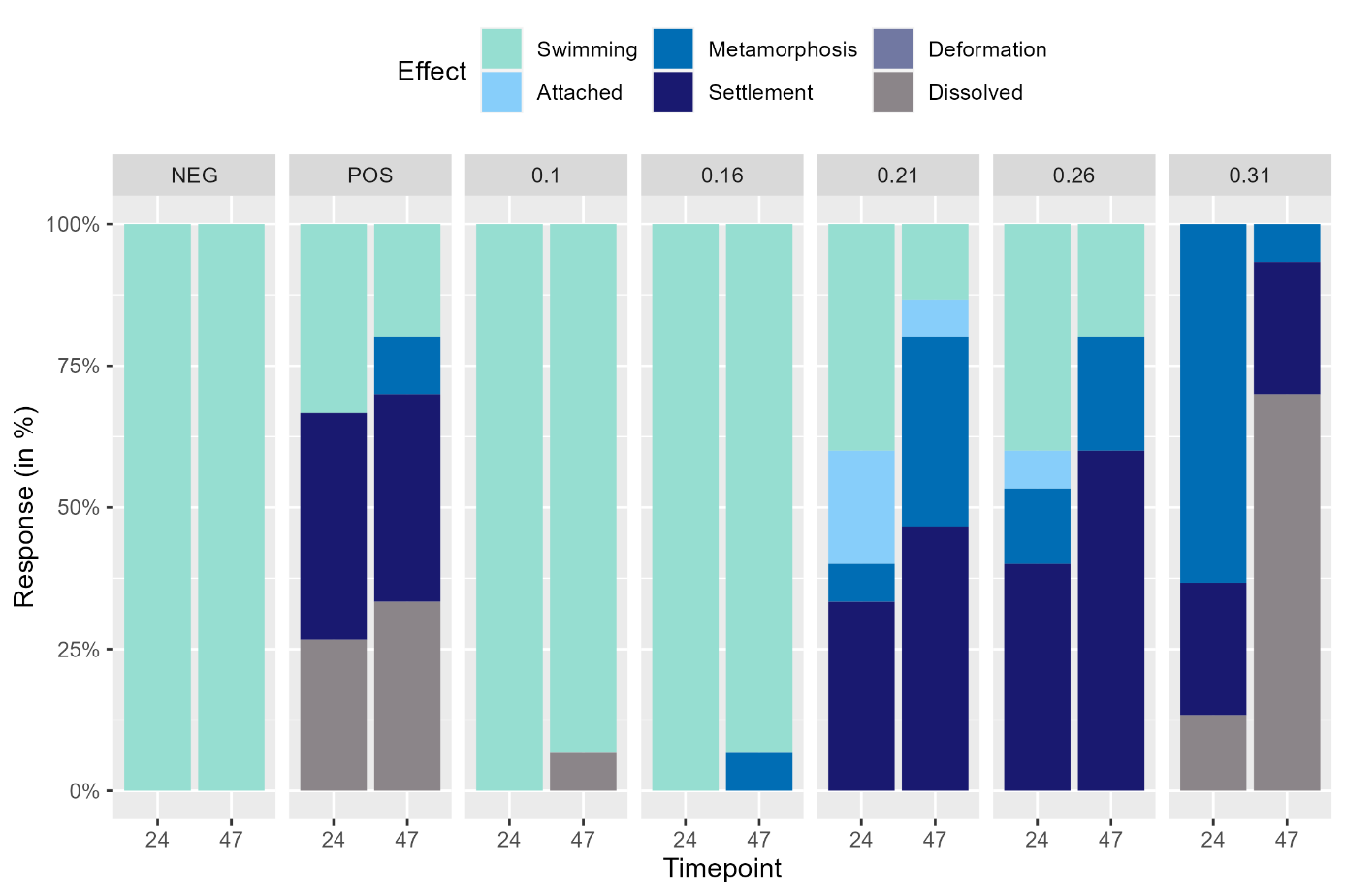


Supplemental Figure 8. Detailed demonstration of the mean effects of CYPRO in different concentrations (0.1, 0.16, 0.21, 0.26 and 0.31 µg cm^-2^) on *Acropora kenti* larvae after 24 and 47 h using 3 replicates with 5 larvae each.


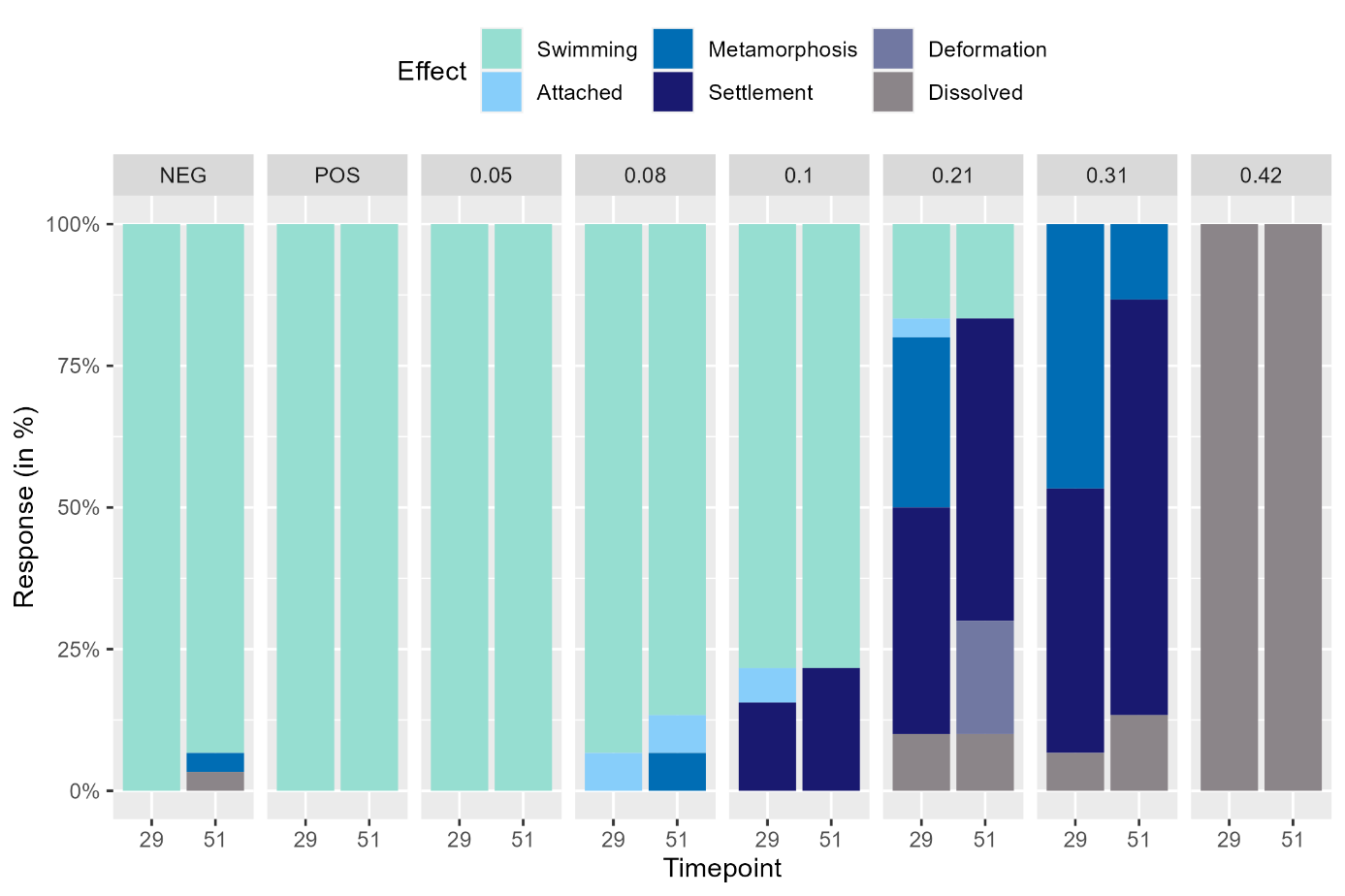
Supplemental Figure 9. Detailed demonstration of the mean effects of CYPRO in different concentrations (0.05, 0.08, 0.1, 0.21, 0.31 and 0.42 µg cm^-2^) on *Acropora millepora* larvae after 29 and 51 h using 3 replicates for the concentrations 0.05; 0.08; 0.31 and 0.42 µg cm^-2^ and 6 replicates for NEG; POS; 0.1 and 0.21 µg cm^-2^.


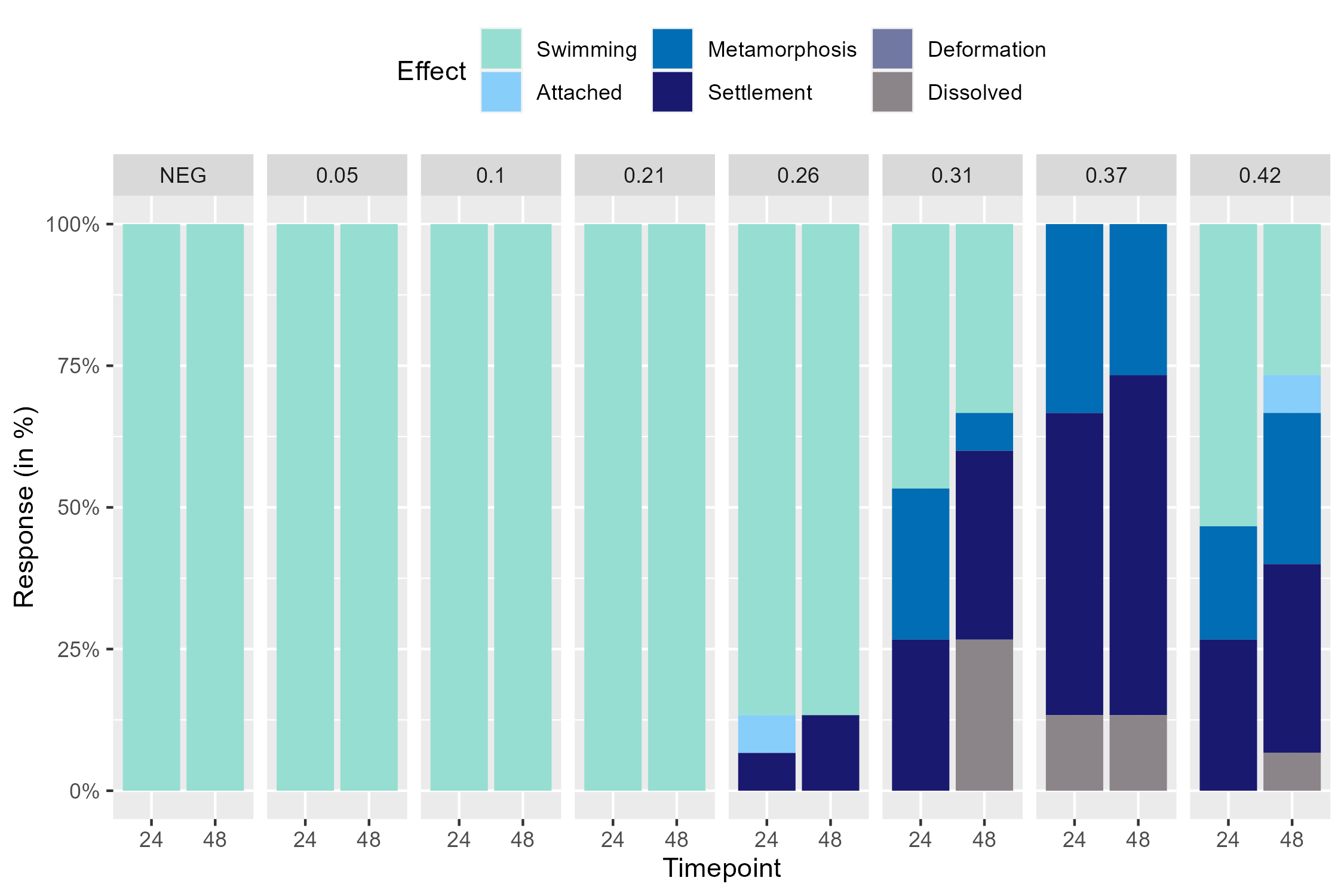


Supplemental Figure 10. Detailed demonstration of the mean effects of CYPRO in different concentrations (0.05, 0.1, 0.21, 0.26, 0.31, 0.37 and 0.42 µg cm^-2^) on *Acropora microclados* larvae after 24 and 48 h 3 replicates with 5 larvae each,

Supplemental Table 1. Detailed information on all conducted settlement experiments. *Plate size refers to the number of wells per plate. ** in one control well were 5 instead of 4 larvae due to a counting error.

| **Species** | **Larval age** | **Plate size*** | **Light settings** | **Larvae per well** | **Positive control** | **CYPRO concentrations [µg cm^-2^]** | **Replicates per concertation** |
| --- | --- | --- | --- | --- | --- | --- | --- |
| *Leptastrea transversa* | stored in AFSW for max 1 week | 12 (size chosen due to reduced availability of 6 well plates during the field trip) | Natural light at the outdoor facility of the Guam University | 5 | live CCA (*Hydrolithon reinboldii*)  freshly collected from Luminao Reef, Guam | 0.14, 0.29, 0.57 and 0.86 | 3 for all tested treatments |
| *Leptastrea purpurea* | stored in AFSW for max 1 week | 12 (size chosen due to reduced availability of 6 well plates during the field trip) | Natural light at the outdoor facility of the Guam University | 5 | live CCA (*Hydrolithon reinboldii*)  freshly collected from Luminao Reef, Guam | 0.14, 0.29, 0.57 and 0.86 | 3 for all tested treatments |
| *Pocillopora acuta* | stored in AFSW for max 3 weeks | 6 | Artificial light composition  (see Sup. Figure 1) | 3 | live CCA (*unknown species*)  cultivated in aquarium facilities | 0.31, 0.42, 0.52 and 0.63 | 3 for all tested treatments |
| *Favia fragum* | stored in AFSW for max 2 weeks | 6 | Artificial light composition  (see Sup. Figure 1) | 5 | live CCA (*unknown species*)  cultivated in aquarium facilities | 0.21, 0.31, 0.42, 0.52 and 0.63 | 6 for all tested treatments |
| *Acropora humilis* | 6 days after spawning | 6 | Artificial light composition  (see Sup. Figure 1) | 5 | none | 0.1, 0.16 and 0.21 | 3 for all tested treatments |
| *Acropora hemprichii* | 7 days after spawning | 6 | Artificial light composition  (see Sup. Figure 1) | 4** | none | 0.05, 0.1 and 0.21 | 3 for all tested treatments |
| *Acropora kenti* | 6 days after spawning | 6 | Artificial light composition  (see Sup. Figure 1) | 5 | live CCA (*unknown species*)  cultivated in aquarium facilities | 0.1, 0.16, 0.21, 0.26 and 0.31 | 3 for all tested treatments |
| *Acropora millepora* | 7 days after spawning | 6 | Artificial light composition  (see Sup. Figure 1) | 5 | live CCA (*unknown species*)  cultivated in aquarium facilities | 0.05, 0.08, 0.1, 0.21, 0.31 and 0.42 | 3 for 0.05; 0.08; 0.31 and 0.42 µg cm^-2^  6 for NEG; POS; 0.1 and 0.21 µg cm^-2^ |
| *Acropora microclados* | 6 days after spawning | 6 | Artificial light composition  (see Sup. Figure 1) | 5 | none | 0.05, 0.1, 0.21, 0.26, 0.31, 0.37 and 0.42 | 3 for all tested treatments |

Supplemental Table 2. Specification of the different categories displayed in the following detailed supplemental figures, described in detail in ^1,2^.

| **Category** | **Definition** |
| --- | --- |
| Swimming | elongated or oval shaped larvae, that are mobile and actively swim or rest |
| Attached | oval shaped larvae that are stable attached to the ground, but have not visibly started their metamorphosis process yet  *note that: stable attachment was controlled after 2 days using a gentle pipette flow* |
| Metamorphosis | Larvae that visibly changed from their larval shape into a roundish or flower-shaped state but are not attached  *Note that if this state is not followed by a subsequent stable attachment, the larvae are not able to develop further and ultimately dies as described in* ^2^ |
| Settlement | Larvae that are stable attached and visibly metamorphosed into a coral recruit (as shown in the top pictures of Figures 3, 4) |
| Deformation | Larvae that exhibit a change in their shape that differs from the uniform roundish, flower-shaped state that is common for metamorphosis  *Note that this was rarely observed after larvae conducted a water-born metamorphosis but were not able to attach* |
| Dissolved | Larvae that started to lose their clear structure and dissolve into the water column, this state indicates the mortality of the larvae |

**SI References**

1. Fiegel, L. J. *et al.* Detailed visualization of settlement and early development in Leptastrea purpurea reveals distinct bio-optical features. *Front. Mar. Sci.* **10**, (2023).

2. Petersen, L.-E. *et al.* Photodegradation of a bacterial pigment and resulting hydrogen peroxide release enable coral settlement. *Sci. Rep.* **13**, 3562 (2023).
